# Family dysfunction and cognitive decline in aging: the “Health, Wellbeing, and Aging” (SABE) longitudinal population-based study

**DOI:** 10.1590/1980-5764-DN-2022-0109

**Published:** 2023-10-23

**Authors:** Diego Ferreira Silva, Juliana Nery Souza-Talarico, Jair Licio Ferreira Santos, Yeda Aparecida Oliveira Duarte

**Affiliations:** 1 Universidade de São Paulo, Faculdade de Enfermagem, São Paulo SP, Brazil.; 2 University of Iowa, School of Nursing, Iowa City, LA, USA.; 3 Universidade de São Paulo, Faculdade de Medicina de Ribeirão Preto, Ribeirão Preto SP, Brazil.; 4 Universidade de São Paulo, Faculdade de Saúde Pública, São Paulo SP, Brazil.

**Keywords:** Family, Aging, Cognitive Dysfunction, Nursing, Família, Envelhecimento, Disfunção Cognitiva, Enfermagem

## Abstract

Stress during aging is not uncommon and dysfunctional family relationships are important sources of stress in the elderly. Considering the potential stressor that family dysfunction represents, it is questioned whether prolonged exposure to dysfunctional family arrangements can contribute to cognitive decline in aging.

**Objective:**

To verify whether family dysfunction is a predictive factor of cognitive decline in aging.

**Methods:**

Secondary study with analysis of existing data from the longitudinal, population-based study “Health, Wellbeing and Aging” (SABE). Data from 791 elderly people from two cohorts of the SABE study between 2006 and 2015 were analyzed. Family dysfunction was assessed using the Apgar family instrument, while cognitive performance was assessed using the Mini-Mental State Examination (MMSE), verbal fluency (animals) and digit length in reverse order. Cognitive decline was measured by the difference in scores in the period between 2006 and 2015.

**Results:**

Approximately 10% of the sample had family dysfunction. The familial Apgar score was not associated with decline on MMSE (p=0.732), verbal fluency (p=0.852) and digit span scores (p=0.718). Scores related to cognition and family functionality, such as age, education, living alone, depression and family Apgar, do not explain cognitive decline.

**Conclusion:**

The findings indicate that family functioning is not associated with cognitive decline in community-dwelling elderly. New studies will be needed to analyze the qualitative characteristics of family relationships in the cognitive performance of the elderly.

## RESUMO.

O estresse ao longo do envelhecimento não é incomum, e as relações familiares disfuncionais constituem fontes importantes de estresse nos idosos. Considerando-se o potencial estressor que a disfunção familiar representa, questiona-se se a exposição prolongada a arranjos familiares disfuncionais pode contribuir para o declínio cognitivo no envelhecimento.

**Objetivo:**

Verificar se a disfunção familiar é um fator preditivo de declínio cognitivo no envelhecimento.

**Métodos:**

Estudo secundário com análise de dados provenientes do estudo longitudinal de base populacional “Saúde, Bem-estar e Envelhecimento” (SABE). Foram analisados dados de 791 idosos de duas coortes do estudo SABE no período entre 2006 e 2015. A disfunção familiar foi avaliada pelo instrumento Apgar familiar, enquanto o desempenho cognitivo foi avaliado pelo Miniexame do Estado Mental (MEEM), fluência verbal (animais) e extensão de dígitos na ordem inversa. O declínio cognitivo foi medido pela diferença dos escores entre 2006 e 2015.

**Resultados:**

Aproximadamente 10% da amostra apresentou disfunção familiar. O escore Apgar familiar não foi associado ao declínio cognitivo pelo MEEM (p=0,732), fluência verbal (p=0,852) e extensão de dígitos ao longo do tempo (p=0,718). Escores relacionados à cognição e funcionalidade familiar, como idade, escolaridade, morar sozinho, depressão e Apgar de família, não explicam o declínio cognitivo.

**Conclusão:**

Os achados mostram que a funcionalidade familiar não está associada ao declínio cognitivo de idosos da comunidade. Novos estudos serão necessários para analisar as características qualitativas das relações familiares no desempenho cognitivo de idosos.

## INTRODUCTION

Impaired family functioning, i.e., the ability of the family to meet and harmonize its essential functions with the identity and tendency of its members and respond appropriately to hazards and opportunities within its social context is associated with declines in independence, autonomy and quality of life of older individuals^1–3^. Older adults from dysfunctional families have a 5-fold greater risk of presenting depressive symptoms compared to those living in functional families^4^. Family dysfunction is also associated with pain, falls and low quality of life^2,5^.

Roughly 10% of older adults have dysfunctional families, a phenomenon associated with individuals who are male, subject to ill-treatment, living alone and dependent for basic and instrumental activities of daily living (ADLs), pain, poor self-rated health, number of falls and osteoporosis^6,7^.

Emotional dysregulation stemming from tense, stressful family relationships, with consequent social isolation, is one of the main outcomes associated with family dysfunction, representing a factor that negatively impacts cognitive health^3,8^. Social interactions, either within the family or with other social actors, promote active aging by allowing the involvement of older adults in activities which represent cognitive challenges and mental well-being. Conversely, social isolation, or lack of social interaction, can be a risk factor for cognitive decline^8^.

The available evidence associating family functioning with cognitive health is scant. However, some studies reveal that indirect measures related to family arrangements, such as marital status, frequency of contact, support for instrumental ADLs, affective expression and conflicting family relationships, are associated with worse cognitive performance. A longitudinal study found that divorced or widowed older adults had greater risk of decline in memory performance and orientation over a 7-year follow-up compared with married individuals^9^. Another study showed that being married was associated with greater satisfaction with life and lower risk of developing mild cognitive impairment or dementia^10^. A population-based study of Chinese older immigrants found that participants with conflicted family relationships had poorer overall cognition and memory performance over a 2-year period than those with unobligated ambivalent relationships^11^. However, these studies centered on the relationship between family typology and interaction, limiting interpretation of the influence of family functioning structure on cognitive performance.

Cross-sectional data from our research group revealed that the poorer the family support, the lower the cognitive performance (global cognition, verbal fluency and digit span)^12^. However, these findings were drawn from a cross-sectional analysis, precluding any meaningful interpretation of the impact of family functioning on the probability of cognitive decline over time^12^.

The objective of the present study was to determine the prevalence of family dysfunction and its association with cognitive decline in aging. The study hypothesis holds that the rate of cognitive decline is associated with poor family functioning, even after controlling for confounding factors such as age, gender, education and depressive symptoms.

Identifying groups vulnerable to cognitive decline can help inform future investigations on risk and protective factors for successful aging and also provide a basis for systematic assessment of family functioning in professional practice, with consequent interventions preventing cognitive decline in older people.

## METHODS

### Study design, setting and participants

**Figure 1:**
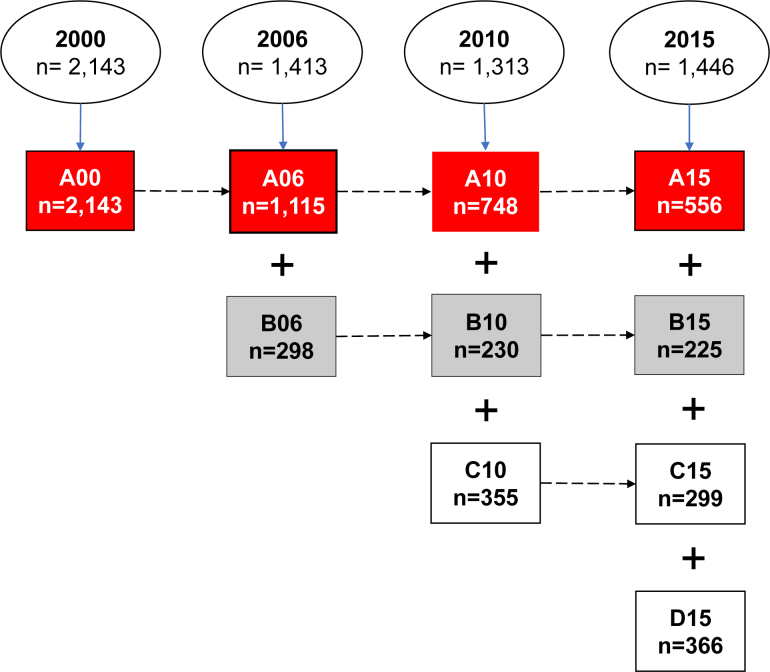
Graphic representation of the cohorts of the “Health, Wellbeing and Aging” (SABE) study, carried out in the city of São Paulo in the years 2000, 2006, 2010 and 2015, with a projection for 2020^13^.

Notes: White circles indicate total sample in 2000 (cohort A), in 2006 (Cohort A06+B06), in 2010 (Cohort A10+B10+C10), and in 2015 (A15+B15+C15+D15). Red boxes indicate cohort A started in 2000 (A00; n=2,143), and its survivors in 2006 (A06; n=1,115), in 2010 (A10; n=748) and in 2015 (A15; n=556). Grey boxes indicate cohort B (60–65 years old) started in 2006 (B06; n=298), and its survivors in 2010 (B10; n=230) and 2015 (B15; n=225). Light grey boxes indicate cohort C (60–65 years old) started in 2010 (C10; n=355) and its survivors in 2015 (C15; n=299). White boxes indicate cohort D (60–65 years old) started in 2015 (D15; n=366) and its survivors in 2015 (C15; n=299).

The present study drew on data from the Brazilian “Health, Wellbeing And Aging” (SABE) study performed in the city of São Paulo, the country's most populous metropolis and home to the largest absolute number of older adults, a group that is highly diverse due to immigration and internal migration^13^. Since 2000, a total of 3,257 Brazilians aged ≥60 years living in the urban area of São Paulo city, comprising four cohorts (A, B, C and D), were included in the study. This population was selected by probability sampling of households registered in census sectors. The households visited were chosen randomly using cluster sampling. SABE participants are reassessed every five years, at which point new cohorts of individuals aged 60–64 years are included (Cohort B in 2006, Cohort C in 2010, Cohort D in 2015). All data were obtained by applying a face-to-face survey at the participants’ homes, conducted by trained interviewers and health professionals (Figure 1). Institutionalized older adults were not included in the SABE study.

Given that family dysfunction was the variable of primary interest in the present study and that assessment of the participants’ family functioning was only included from 2006, the population universe of the present investigation comprised participants from cohorts A06 and B06 (nA06+nB06=n=1,413; Figure 1).

**Figure 2:**
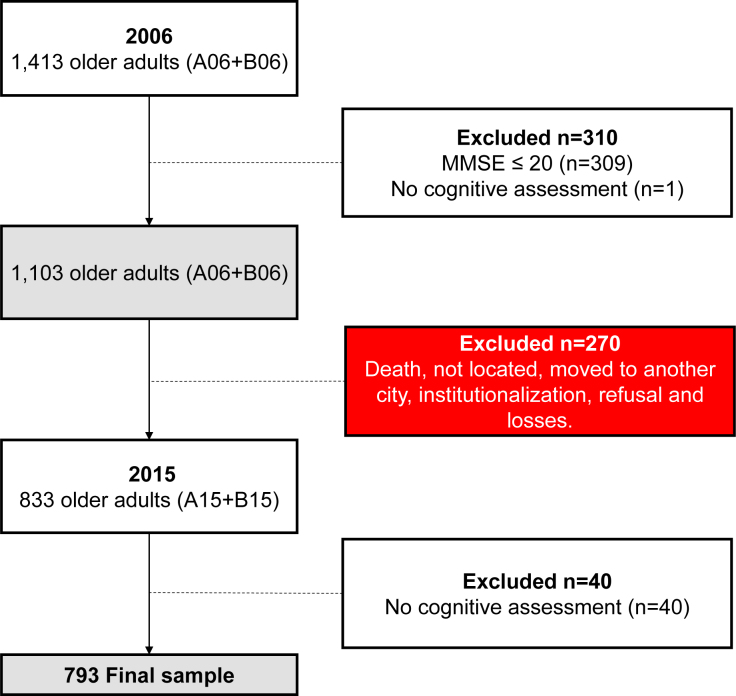
Flow diagram showing sample composition. Gray boxes indicate the sample size in 2006 (baseline for the current study) and the sample in 2015, while the red box indicates missing cases during follow-up (n=270).

Of the 1,413 records of older adults (≥60 years) from cohorts A06 and B06, exclusions were made according to the following criteria:

- A score <20 on the Mini-Mental State Examination — MMSE (n=309);
- No cognitive evaluation performed in 2006 (n=1);
- A move to another state, transfer to a long-term care facility, refusal to participate, or death (n=270); and
- No cognitive assessment in 2015 (n=40; Figure 2).

Application of the criteria led to the exclusion of 620 patients, giving a final sample of 793 patients (Figure 2).

The study was approved by the Ethics Committee for Research in Humans of School of Public Health of the University of São Paulo (#COEP/23/10), São Paulo, Brazil. All participants provided informed written consent and information on their identity was anonymized.

### Measures

#### Cognitive performance

Cognitive decline was measured based on the difference in test scores between the baseline assessment in 2006 and final assessment in 2015. Score difference (delta) was used for the following cognitive tests:

- MMSE — comprising question items grouped into seven categories: temporal orientation, spatial orientation, immediate memory, attention and calculus, delayed recall (long-term memory), language and visuoconstructive ability, adapted for use in Brazil.^14^ Cut-off points varied according to the educational level of the respondent (≤20 points for illiterate subjects; ≤25 points for individuals with 1–4 years of education; ≤26 points for 5–8 years; ≤28 points for 9–11 years; and ≤29 points for individuals with >11 years of formal education)^14,15^.
- Verbal fluency (animals) — assesses the semantic component of verbal fluency. Participants must say as many names of animals as they can within 1 minute. The total number of animals produced represents the score on the test. Scores below the cut-off values according to education indicate a cognitive deficit (cut-off score ≤9 for illiterate subjects; ≤12 for 1–7 years of education; ≤13 for >7 years of education)^14,15^.
- Backward digit span^16^ — measures working memory performance and, in the SABE study, consists of repeating five random digits in reverse order read aloud by the examiner at a rate of one1 per second. Each digit repeated correctly scores 1, giving a maximum score of 5^16,17^.

The cognitive decline variable for each of the tests used classifies the participant into two categories:

- Cognitive decline absent — comprising individuals whose test score remained stable or improved between assessments performed in 2006 and 2015.
- Cognitive decline present — comprising individuals whose scores on the test worsened between assessments performed in 2006 and 2015.

#### Family support

The family Apgar^18^, a valid instrument for Brazilian older adults^19^, evaluated the family functionality components of adaptability, partnership, growth, Affection, and resolve using five questions to reflect participants’ views on the functional status of their family. The participants rated their satisfaction with family partnership, empathy, support, care, and interaction for each question on a scale from 0 (never) to 4 (always). The total score ranges from 0 to 20 points and is calculated as the sum of points on each question. Scores ≤12 indicate family dysfunction.

### Covariates

The following variables were analyzed:

- Sociodemographic (i.e., gender, age, education [years of study], race, marital status, income (Brazilian Institute of Geography and Statistics — IBGE criteria, and retirement status);
- Health status (e.g., self-reported hypertension, heart disease, diabetes, chronic obstructive pulmonary disease, stroke, medication use, current smoking and alcohol abuse, body mass index [measured], self-rated health — excellent, fair, poor);
- Family structure (i.e., number of people living with the participant, living alone);
- depression symptoms (i.e., geriatric depression scale — GDS); and
- Frailty (using the components proposed by Fried et al.). The frailty phenotype was obtained through the following measurable components: unintentional weight loss, handgrip strength, fatigue, reduced walking speed, and low physical activity. Positive answers scored one point. The frailty classification followed Fried et al.'s proposal (i.e., score ≥3 frail, score of 1–2 pre-frail and 0=non-frail)^20^.

### Data analysis

Descriptive statistics of absolute and relative frequencies, measures of central tendency and dispersion were used to characterize the sample according to dependent, independent and confounding variables. Student's t-test for comparing means and Chi-square for frequencies were used to compare sociodemographic, health and family characteristics between groups (current sample x exclusion/missing). Repeated measures were used to analyze changes on cognitive scores from 2006 to 2015 between functional and dysfunctional families. Multivariate logistic regression models were used to analyze associations of cognitive decline (absent x present) as the dependent variable and family functioning (family Apgar score). The regression models were adjusted for age, education, living alone and GDS score. The level of significance adopted for statistical tests was 0.05 for a 95% confidence interval.

## RESULTS

The sample predominantly comprised participants who were female, low-educated, white, married and with a low-to-middle monthly income (Table 1). Regarding health, most participants had hypertension, cardiovascular disease and diabetes. The rate of smoking and alcohol misuse was low. Depressive symptoms were low overall, as was the percentage of frail individuals, in the evaluation of the frailty syndrome. Most respondents rated their health as fair or excellent (Table 1). For cognitive performance, participant scores were within cut-off values (Table 1). The majority of the participants lived with others and family dysfunction was detected in around 10% (Table 1). With the exception of gender and self-rated health, no significant differences were found between participants that remained in the study until 2015 and those who did not (losses) (Table 1).

**Table 1:** Participant characteristics, health status, cognitive performance and family functioning.

		SABE sample 2006 n=1,413		Current study sample n=793	Exclusion/missing n=620	p-value*
		Mean (±SD) n (%)	Min–Max	Mean (±SD) n (%)	Mean (±SD) n (%)	
Age (years)		69.1	60–97	69.2	69.0	0.207†
Education (years)		4.7	0–17	4.97	4.64	0.063†
Gender (% female)		55.6	--	55.4	66.7	0.004‡
Race (%)						
	White	64.6	--	64.2	65.4	0.663‡
	Brown	19.1	--	20.2	16.3	
	Black	6.62	--	6.27	7.48	
	Indigenous	0.57	--	0.62	0.45	
	Yellow	3.05	--	3.13	2.87	
	Not reported	6.03	--	5.48	7.40	
Marital status (%)						
	Divorced	2.26	--	1.67	3.71	0.105‡
	Separated	5.50	--	5.96	4.38	
	Widow(er)	27.9	--	25.8	33.2	
	Married	55.6	--	57.5	50.8	
	Cohabiting	4.44	--	4.33	4.70	
	Single	4.13	--	4.54	3.12	
	No response given	0.11	--	0.16	0.00	
Income (%)						
	No income	5.08	--	5.58	4.02	0.770‡
	≤1 minimum wage	20.8	--	17.7	27.4	
	1–2 minimum wages	24.7	--	27.0	19.9	
	2–3 minimum wages	16.6	--	16.9	15.9	
	3–5 minimum wages	17.1	--	17.5	16.2	
	>5 minimum wages	15.6	--	15.2	16.4	
	Retired (% yes)	730 (72.0)	--	522 (71.2)	208 (74.0)	
Self-rated health						
	Hypertension (% yes)	62.1	--	62.9	60.1	0.482‡
	Heart disease (% yes)	21.1	--	22.4	17.9	0.160‡
	Diabetes (% yes)	20.4	--	20.5	20.2	0.925‡
	COPD (% yes)	11.9	--	12.2	11.2	0.692‡
	Cerebrovascular stroke (% yes)	5.81	--	7.08	2.65	0.005‡
	Use of medication (% yes)	90.4	--	91.3	88.1	0.254‡
	Smoker (% yes)	48.4	--	52.4	39.3	0.176‡
	Alcohol misuse (% yes)	1.42	--	1.85	0.35	0.087‡
	Body mass index	26.7	15–51	26.4	27.3	0.080‡
	Geriatric Depression Scale (GDS)	2.39	0–15	2.28	2.35	0.574‡
Frailty						
	Non-frail	54.9		52.9	59.8	0.152‡
	Pre-frail	40.4		42.4	35.7	
	Frail	4.58		4.63	4.47	
Self-rated health (% yes)						
	Excellent	47.9	--	46.4	51.7	0.263‡
	Fair	45.0	--	45.7	43.1	
	Poor	6.99	--	7.73	5.18	
	Mini-mental state examination	26.1	21–30	26.0	26.3	0.100†
	Verbal fluency (animals)	12.	1–29	12.1	12.2	0.995†
	Backward digit span	4.1	0–5	4.1	4.3	0.085†
	Number of people living with respondent	2.99	1–14	3.00	2.97	0.864†
	Living alone (% yes)	100	--	86.9	13.4	0.888‡
	Family apgar (score)	17.5	0–20	17.4	17.9	0.616†
	Family dysfunction (% yes)	10.1	--	10.4	9.32	0.166‡

Abbreviation: SABE, Health, Wellbeing, and Aging; SD, standard deviation; COPD, Chronic Obstructive Pulmonary disease. Notes:

* p≤0.05 statistical significance;

† student t-test;

‡ chi-square.

### Cognitive decline over a ten-year period and family functioning

**Figure 3:**
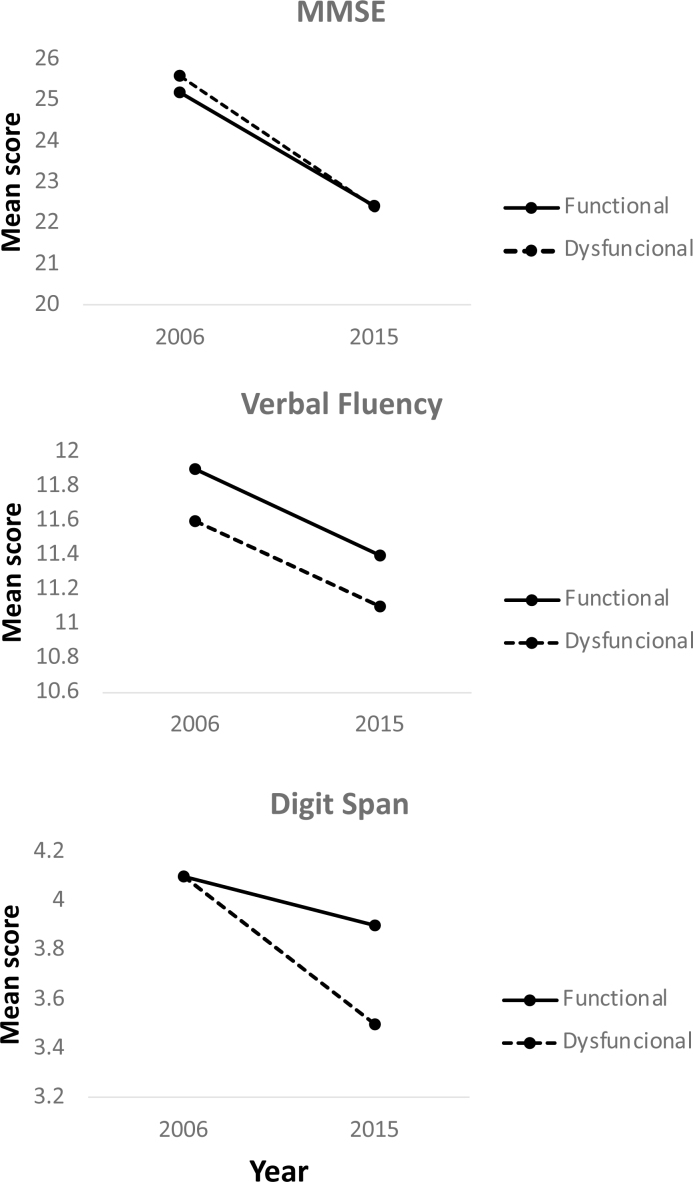
Mean scores of Mini-Mental State Examination, verbal fluency and digit span in individuals with functional and dysfunctional families.

Abbreviation: MMSE, Mini-Mental State Examination. Note: Changes in cognitive score means from 2006 to 2015.

There was a significant decline in mean score for the MMSE (p<0.001), verbal fluency (p=0.002) and digit span tests (p<0.001; Figure 3A). The magnitude of these declines on the MMSE (p=0.732), verbal fluency (p=0.852) and digit span (p=0.718), however, proved similar for individuals with and without family dysfunction (Figure 3B, 3C and 3D).

### Association between family functioning and cognitive performance

In the multivariate linear regression analysis, no influence of Family Apgar score on cognitive decline on the MMSE, verbal fluency or digit span tests was evident (Table 2).

**Table 2:** Logistic regression coefficients between decline on cognitive tests and Apgar scores, adjusted for covariates (age, education, living alone, Geriatric Depression Scale score).

Variables	MMSE (n=319)			Verbal fluency (n=568)			Digit span (n=330)		
	OR	p	95%CI Min–Max	OR	p	95%CI Min–Max.	OR	p	95%CI Min–Max.
Age	1.01	0.515	0.970–1.060	0.98	0.139	0.956–1.006	1.00	0.955	0.961–1.041
Education	1.04	0.199	0.976–1.116	1.05	0.034	1.004–1.117	1.02	0.536	0.953–1.094
Living alone	0.74	0.425	0.360–1.543	1.087	0.739	0.660–1.790	0.96	0.922	0.444–2.086
GDS score	0.96	0.4494	0.882–1.063	0.962	0.362	0.884–0.046	1.02	0.709	0.931–1.109
Apgar score	0.97	0.502	0.922–1.040	0.961	0.124	0.913–1.011	1.04	0.079	0.994–1.106

Abbreviations: GDS, Geriatric Depression Scale; MMSE, Mini-Mental State Examination; OR, odds ratio; CI, Confidence Interval. Notes: *p≤0.05 indicates statistical significance.

## DISCUSSION

In the present study, whose objective was to determine the prevalence of family dysfunction and its association with cognitive decline in aging, only 10% of the sample had family dysfunction. These findings corroborate the results of other studies, reporting prevalence rates of family dysfunction ranging by 9–10% in the samples investigated^21,22^.

In aging, family dysfunction is characterized primarily by the interaction between low social support and impaired family bonds^23^. Moreover, occupational, economic and functional challenges can compromise family harmony during aging^22^. Social support, such as financial assistance, transport and help with domestic chores, are essential for the well-being of older people^24^. In the present study, most of the participants were classified as having a moderate economic level, perhaps explaining, albeit in part, the low rate of family dysfunction.

Family support should be bi-directional, i.e. older adults are supported by family members and, in turn, help with activities within the family structure (e.g. taking care of grandchildren, household tasks, informal work activity to boost income). Close contact with the older person allows family members to pinpoint physical, functional or emotional changes quickly^25^. In the present study, most of the participants lived with other family members, another factor possibly explaining the low rate of family dysfunction.

Recent studies suggest that caring for an elderly person can have negative impacts on the caregiver's physical and mental health, especially when the care is related to some form of dementia, which can lead to high levels of stress, depression, anxiety, pain, fatigue and insomnia, which can even present negative effects in the future for the elderly^25^. However, in the study, a low rate of family dysfunction was observed, which may suggest that caring for the elderly can have positive effects in some families. A review that aimed to identify the positive aspects of caring for someone with dementia listed effects such as emotional satisfaction, a sense of purpose and better quality of life^26^.

However, contradicting the main hypothesis of the present study, no effect of family functioning on cognitive decline was detected. Although significant declines in scores on the MMSE, verbal fluency and digit span tests were seen over the follow-up period, these declines proved similar for individuals with and without family dysfunction. In addition, the family Apgar score had no influence on the odds of cognitive decline over the ten-year follow-up, i.e. according to participant perceptions, family functioning (satisfaction with partner, empathy, support, care and interaction with family members) had no influence on the risk of cognitive decline over ten years.

Echoing these findings, a previous cross-sectional study of 2,052 older Brazilians (mean age=70.8 years) failed to observe a significant association between family dysfunction, as measured by the Apgar, and cognitive performance^27^. Also, in a longitudinal study conducted in Japan (mean age of participants around 73 years), the authors found that family support had no influence on cognitive performance^27^. However, the support of neighbors and friends was associated with better cognitive performance, highlighting that this type of support can be a protective factor against cognitive decline, neutralizing the influence of family relationships^28^.

However, the results of the cross-sectional Population Study of Chinese Elderly in Chicago, involving a sample of Chinese immigrants, revealed that detached, commanding, conflicted, and tight-knit relationships were associated with poorer performance in episodic memory, working memory and MMSE^11^. However, unlike the present study, the authors analyzed family relationships rather than family functioning, which potentially explains the disparities in findings. In a cross-sectional analysis of the SABE cohort which included only older adults aged 60–64 years, functional family support, but not structural support (number of individuals living with older adult), was found to be associated with higher scores on the MMSE, verbal fluency and digit span tests^12^. Moreover, the authors found that, unlike receiving social support, providing the community with support services was associated with better cognitive performance^12^. It is important to point out, however, that the population in question was younger than that of the present study, and participants with low cognitive performance were not excluded. The cross-sectional design precludes analysis of cause and effect, allowing the risk of reverse causality in which cognitive impairment may have already existed in the sample of older individuals, thereby explaining the dysfunctional family relationship. Cognitive impairment can significantly impact functional independence of older adults and of the family unit. The present study hypothesis holds that family dysfunction leads to cognitive decline. Thus, excluding individuals with low cognitive performance in 2006 reduced the likelihood that family burnout was already present in the population study.

The absence of an association in this study suggests that the protective role of family relationships on the cognitive health of older individuals might only be significant in young-old. Cognitive reserves decrease in older age, yet with synergic factors, such as social or family interactions, this decline produces little or no effect^29^. In addition, the present study was conducted in a cohort of older Brazilians with average income. Consequently, the sociodemographic and cultural characteristics of Latino populations can explain why family dysfunction did not represent a risk factor for cognitive decline in this study. Factors moderating this relationship, such as resilience stemming from a positive outlook on life and its challenges, might also attenuate the negative effect of family dysfunction. Corroborating this theory, a Brazilian study showed that most of the older adults investigated (n=84.9%) had a high score on an instrument measuring resilience and a positive association with cognitive performance^30^. This lends credence to the hypothesis that the absence of a significant association between family dysfunction and cognition in the present study participants is explained by the resilience and perception this group has to overcome adversities arising in their lives, along with the negative impact of family dysfunction over time.

The inconsistency in the findings of the present study *versus* data from the literature indicates the need for further longitudinal studies encompassing both quantitative and qualitative data. Future studies can analyze not only family functioning but also the characteristics defining the relationships built in living with the older adults, as well as the moderating role of resilience, coping and spirituality in the relationship between family functioning and cognitive decline. Identifying the factors which preserve cognitive reserve in aging is a crucial element for promoting healthy, active aging.

### Study limitations

Given the study entailed secondary analysis of existing data, some methodological limits in interpreting the results should be noted. The first of these limitations relates to the cognitive assessment. The use of specific tests for assessing abilities such as information processing speed, attention, executive function and short-term memory, which decline with age, may yield different results to those reported. An assessment of functional capacity for ADLs in all participants would also add to the analyses carried out, enabling individuals to be classified according to cognitive impairment as opposed to only performance decline over time. The aim of the SABE study was to identify the living and health conditions of the older population in the city of São Paulo, explaining the availability of more global cognitive data such as that obtained using the MMSE and verbal fluency tests. Although studies that analyze existing data represent an innovative cost-effective approach, drawbacks in terms of measuring the variables of interest of the secondary study may introduce bias and, hence, influence results. Another important point involves losses over the long follow-up period. In view of the low difference in cognitive performance between 2006 and 2015, the reduction in sample size may have influenced the power of statistical tests to identify significant differences.

Notwithstanding these limitations, the present study is the first to analyze the influence of family relationships on cognitive decline using longitudinal data from a probability sample of community-dwelling older adults. The SABE study is the only longitudinal population-based study in São Paulo city which has been running for more than 20 years. The methodological rigor with which the data are collected and organized confer greater robustness, relevance and originality to the evidence reported.

In conclusion, although family dysfunction is prevalent in aging and global cognitive performance, and verbal fluency and working memory declined over the ten-year follow-up, the present study did not find an association between cognitive decline and family functioning. The influence of factors moderating the relationship between family functioning and cognitive performance should be explored in future studies to determine which factors enhance and which impair cognition during aging.
